# An mRNA-LNP Vaccine Encoding the E^rns^-E1-E2 Polyprotein Confers Complete Protection Against Bovine Viral Diarrhea Virus in Mice

**DOI:** 10.3390/ani16203313

**Published:** 2026-10-21

**Authors:** Zongzhe Yue, Yanmin Li, Hongjun Yang, Shiao Bian, Xiaoai Meng, Lixiang Zhao, Zhidong Zhang

**Affiliations:** 1 Key Laboratory of Veterinary Medicine of Sichuan Provincial Universities, College of Animal and Veterinary Sciences, Southwest Minzu University, Chengdu 610041, China; 15738535923@163.com (Z.Y.); liyanmin@swun.edu.cn (Y.L.); 19856235652@163.com (S.B.); 18386117571@163.com (X.M.); 2 Shandong Academy of Agricultural Sciences, Jinan 250100, China; yanghongjun166@163.com; 3 Suzhou Medical College, Soochow University, Suzhou 215123, China; 4 Suzhou HealiRNABiotechnology Co., Ltd., Suzhou 215028, China

**Keywords:** bovine viral diarrhea virus, mRNA vaccines, E glycoprotein, protective efficacy

## Abstract

Bovine viral diarrhea virus (BVDV) remains a major barrier to sustainable livestock development. Conventional BVDV vaccines are limited by inherent drawbacks such as potential virulence reversion and insufficient protective efficacy, which severely hamper global BVDV eradication efforts. mRNA vaccine technology, characterized by high expression efficiency, a favorable safety profile, and scalable manufacturing, offers a promising platform for next-generation BVDV vaccine development. In this study, we developed an mRNA-LNP vaccine candidate (E^rns^/E1/E2 vaccine) encoding the full-length structural glycoproteins E^rns^, E1 and E2, and systematically evaluated its immunogenicity and protective efficacy in BALB/c mice. The E2 extracellular domain-human IgG1 Fc fusion mRNA vaccine (E2/IgG1 vaccine) and a commercial inactivated BVDV vaccine served as controls. Our results demonstrated that the E^rns^/E1/E2 vaccine elicited significantly higher neutralizing antibody titers (log_10_NT_50_ = 2.50 ± 0.082) and lymphocyte proliferation responses (stimulation index = 6.927 ± 0.145) than both control vaccines at 28 days post-primary vaccination (two weeks post-booster). Notably, no viral RNA was detected in any tissue or blood samples from E^rns^/E1/E2-immunized mice at any time point following BVDV challenge, indicating complete immune protection against viral infection, whereas viral loads were consistently detected in multiple tissues of both control groups. These findings establish the E^rns^/E1/E2 vaccine as a highly promising BVDV vaccine candidate and provide critical experimental support for advancing mRNA-based BVDV vaccine development.

## Simple Summary

Bovine viral diarrhea virus (BVDV) is a major obstacle to sustainable cattle farming worldwide, but current vaccines are limited by the risk of regaining virulence and by insufficient protective efficacy, which together hamper global efforts to eradicate the disease. To solve this problem, we developed a messenger RNA vaccine delivered in lipid nanoparticles that encodes the three full-length structural glycoproteins (E^rns^, E1 and E2) of BVDV and evaluated its immune response and protective efficacy in mice, comparing it against both a second messenger RNA candidate and a commercial inactivated vaccine. The new vaccine elicited significantly higher neutralizing antibody titers and stronger lymphocyte proliferation responses than either control. Most importantly, when vaccinated mice were later exposed to the live virus, no trace of the virus was found in any of their tissues or blood, demonstrating the new vaccine provided complete protection, while the comparison groups still carried the virus in multiple tissues. These findings show that this messenger RNA vaccine is a highly promising candidate for protecting cattle against this devastating disease, potentially offering a safer, more effective tool to protect cattle and support the global effort to control and eliminate BVDV.

## 1. Introduction

Bovine viral diarrhea virus (BVDV) represents one of the most economically impactful pathogens affecting the global cattle industry, causing reproductive disorders, respiratory disease, enteritis, and persistent infection in susceptible animals [1,2,3]. BVDV is a spherical, enveloped, single-stranded, positive-sense RNA virus belonging to the genus *Pestivirus* within the family *Flaviviridae* [4,5]. Based on nucleotide sequence variations, BVDV is classified into three genotypes (BVDV-1, BVDV-2 and BVDV-3), each comprising several subgenotypes [6,7,8,9,10]. Of these, BVDV-1 is the predominant epidemic strain in China. The full-length genome of BVDV is approximately 12.3 kb, encoding four structural proteins: a nucleocapsid core protein (C) and three envelop proteins (E^rns^, E1 and E2) and eight non-structural proteins (Npro, P7, NS2, NS3, NS4A, NS4B, NS5A and NS5B) [11,12]. The BVDV genome is translated as a single polyprotein, which is co- and post-translationally cleaved into individual mature proteins by host signal peptidases [13]. The envelope glycoproteins are encoded within the N-terminal region of the polyprotein in the order E^rns^-E1-E2. Processing of this region begins with signal peptidase-mediated cleavage between the capsid protein and the nascent E^rns^-E1-E2 precursor, followed by cleavage at the C-terminus of E2. The E^rns^-E1-E2 precursor is then rapidly cleaved to generate the E^rns^-E1 intermediate and E2; subsequently, the E^rns^-E1 intermediate is further processed to release the individual Erns and E1 proteins [14,15].

Vaccination represents the cornerstone of the BVDV prevention and control programs. Traditional BVDV vaccines, including modified live vaccines (MLVs) and inactivated vaccines, have been widely used for decades and can induce protective immune responses in vaccinated animals [16,17]. However, they are limited by inherent drawbacks: MLVs carry the risk of virulence reversion and lack DIVA (differentiation of infected from vaccinated animals) compatibility, whereas inactivated vaccines often induce weaker and shorter-lived immunity, necessitating multiple doses and adjuvants [18,19,20]. These limitations severely hamper global BVDV eradication efforts. underscoring the urgent need for novel, safe, and highly effective BVDV vaccines. mRNA vaccine technology offers a promising platform for next-generation BVDV vaccine development, owing to its high expression efficiency (achieved through sequence optimization), excellent safety profile (free from risks of virulence reversion and genomic recombination), and scalability for manufacturing [21,22,23,24,25].

The envelope glycoproteins of BVDV (E^rns^, E1, and E2) play critical roles in viral entry, host immune evasion, and induction of protective immune responses. E2 is the major immunodominant glycoprotein, harboring most of the identified neutralizing epitopes. E1 is essential for forming functional heterodimers with E2 to mediate viral fusion. Erns possesses RNase activity and also contains neutralizing epitopes that contribute to protective immunity. In this study, we designed two mRNA vaccine candidates targeting BVDV envelope proteins: (i) E^rns^/E1/E2 mRNA vaccine encoding the full-length E^rns^-E1-E2 protein as the primary experimental candidate; (ii) E2/IgG1 mRNA vaccine encoding the E2 extracellular domain fused to human IgG1 Fc to enhance antigen presentation, which served as a control candidate. We systematically evaluated the immunogenicity and protective efficacy of both candidates in a BALB/c mouse model, in direct comparison with a commercial inactivated BVDV vaccine. Our results demonstrated that the E^rns^/E1/E2 mRNA-LNP vaccine elicited significantly stronger humoral and cellular immune responses and conferred superior protection against BVDV challenge compared to both the E2/IgG1 candidate and the inactivated vaccine. This study provides important experimental evidence and novel insights for the development of next-generation mRNA-based BVDV vaccines.

## 2. Materials and Methods

### 2.1. Cells and Virus

The MDBK cells were maintained in our laboratory, and the HEK-293T cells were stored at Suzhou HealiRNA Biotechnology Co., Ltd. (Suzhou, China). The BVDV-1a strain used in this study was provided by our laboratory. The BVDV-1a strain used in this study was kept in our laboratory, and the strain was identified as BVDV-1a by phylogenetic analysis of the E2 sequence.

### 2.2. Construction and Preparation of BVDV mRNA Vaccines

The target gene of BVDV-1a was amplified by PCR and cloned into the pUC57 vector (Thermo Scientific, Vilnius, Lithuania) to generate recombinant plasmids, which were then transformed into *Escherichia coli* strains. The recombinant plasmids were extracted using an endotoxin-free plasmid extraction kit (Omega Bio-tek, Inc., Norcross, GA, USA) and verified by sequencing (GENEWIZ, Suzhou Jinweizhi Biotechnology Co., Ltd., Suzhou, China). The correctly verified recombinant plasmid was linearized using restriction enzymes and purified to serve as the DNA template for in vitro transcription (IVT). The IVT was performed by a co-transcriptional capping method with the cap analog of CAG Trimer (Vazyme, Nanjing, China). Uridine was completely replaced by the modified nucleoside, with the modification being N1-Methyl-Pseudouridine (N1-ψ). The IVT yield reached 8.5 mg/mL, and the polyA sequence was directly constructed on the plasmid backbone mRNA, which was synthesized via IVT reaction using T7 RNA polymerase, with the addition of cap analog, nucleotides, and other required components. Following IVT, the residual DNA template was degraded by adding DNaseI, and the synthesized mRNA was purified by LiCl precipitation. Under the adopted IVT reaction conditions, the level of dsRNA generated during IVT is generally low and was not quantified. To verify the expression activity of the synthesized mRNA, HEK-293T cells were transfected with each mRNA construct using Lipofectamine 3000 transfection reagent (Thermo Scientific, Vilnius, Lithuania) according to the manufacturer’s protocol. At the predefined time points post-transfection, total cellular proteins were extracted using Beyotime Western and IP Cell Lysis Buffer (Beyotime Biotechnology, Shanghai, China), and the expression of the target protein was verified by Western blot.

To prepare mRNA–LNP, ionizable lipids (SM-102) (Sinopeg, Xiamen, China), 1,2-distearoyl-sn-glycero-3- phosphocholine (DSPC), cholesterol (Avt-avt, Shanghai, China), and 2-dimyristoyl-rac-glycero-3-methoxypolyethylene glycol-2000 (PEG2K-DMG) (Sinopeg, Xiamen, China) were dissolved in anhydrous ethanol at a molar ratio of 50:10:38.5:1.5 (nitrogen to phosphorus ratio = 5.67) to form the organic phase. mRNA was dissolved in 20 mM citrate-sodium citrate buffer (pH 4.0) to form the aqueous phase. The two phases were mixed using a microfluidic device at a flow rate ratio of 1:3 (organic:aqueous) and a total flow rate of 12 mL/min. The resulting mRNA-LNP suspension was diluted 10-fold with phosphate-buffered saline (PBS, pH 7.4) and concentrated by ultrafiltration to remove residual ethanol and exchange the buffer into PBS. The particle size and polydispersity index (PDI) of the mRNA-LNPs were measured by dynamic light scattering (DLS) using an Omec nanoparticle size and zeta potential analyzer (NS-90Z) instrument (Omec Instruments Co., Ltd., Zhuhai, China). The encapsulation efficiency was determined using the RiboGreen^®^ assay kit (Invitrogen, Eugene, OR, USA) according to the manufacturer’s instructions. The expression activity of the mRNA-LNPs was verified by transfection into HEK-293T cells as described above, followed by Western blot analysis of target protein expression.

### 2.3. RNA Extraction and cDNA Synthesis

Total RNA was extracted from BVDV-infected MDBK cells and mice tissues using TRIzol reagent (Life-iLab, Shanghai, China), and cDNA was synthesized via reverse transcription using the ExonScript RT SuperMix kit (ExonGen, Chengdu, Sichuan, China).

### 2.4. Preparation of Recombinant BVDV E2 Protein

The recombinant plasmid pET-28a (+)-E2 expressing BVDV E2 was constructed and maintained by our laboratory. This plasmid pET-28a (+)-E2 was transformed into *Escherichia coli* BL21 competent cells (Sangon Biotech, Shanghai, China) for protein expression. Transformed bacteria were cultured in Luria–Bertani (LB) medium containing 50 μg/mL kanamycin at 37 °C with shaking at 220 rpm until the OD_600nm_ reached 0.6–0.8. Protein expression was induced by the addition of isopropyl β-D-1-thiogalactopyranoside (IPTG) (Sangon Biotech, Shanghai, China) to a final concentration of 0.6 mM. The E2 recombinant protein was subsequently purified using a His Cap 6FF protein purification column (Smart-Lifesciences, Changzhou, Jiangsu, China) according to the manufacturer’s instructions. The quality and identity of the purified BVDV E2 recombinant protein were verified by SDS-PAGE and Western blotting. Briefly, the denatured protein separated by SDS-PAGE was transferred onto a PVDF membrane, which was then blocked with TBST containing 5% milk for 2 h at room temperature. The membrane was incubated overnight at 4 °C with a BVDV E2 monoclonal antibody diluted 1:500, followed by incubation with an HRP-conjugated goat anti-mouse secondary antibody (Thermo Fisher Scientific Inc., Waltham, MA, USA) diluted 1:3500. Protein bands were visualized using the ECL Western blotting substrate (Affinity Biosciences, Liyang, Jiangsu, China) via chemiluminescence.

### 2.5. Detection of E2 Antibody by Indirect ELISA

The purified BVDV E2 recombinant protein was diluted to 0.25 μg/mL with TBS buffer. A volume of 100 μL of the diluted BVDV E2 recombinant protein was added to each well of a 96-well ELISA microplate (Servicebio, Wuhan, Hubei, China) and incubated overnight at 4 °C. The plates were washed three times with 200 μL PBST and blocked with 5% non-fat milk in PBST for 1 h at 37 °C. Serum samples (50 μL/well) were added in triplicate and incubated at 37 °C for 1 h. After three washes with PBST, 100 μL of HRP-conjugated goat anti-mouse IgG secondary antibody (diluted 1:5000 in PBST) was added to each well and incubated at 37 °C for 1 h. After five washes with PBST, 100 μL of TMB substrate (Biosharp, Hefei, Anhui, China) was added to each well, and the plates were incubated for 15 min at room temperature in the dark. The reaction was stopped by adding 50 µL per well of 2 M H_2_SO_4_, and the absorbance was measured at OD_450nm_ using a microplate reader (PerkinElmer, Springfield, IL, USA).

### 2.6. Animal Experiments

Specific-pathogen-free (SPF) female BALB/c mice (5 weeks old) were purchased from Chengdu Dossy Experimental Animals Co., Ltd. (Chengdu, Sichuan, China). Animal experiments were conducted strictly in accordance with the guidelines and regulations of the P.R. China on the use and care of laboratory animals. The protocols for experiments were approved by the University Animal Care and Ethics Committee (SMU-202601251; 2 January 2026). Mice were housed in a temperature-controlled environment (22 ± 2 °C) with a 12 h light/dark cycle and free access to sterilized food and water. After 1 week of acclimatization, mice were randomly divided into four groups (17 mice per group): (1) inactivated vaccine group: subcutaneously immunized with 0.5 mL of a commercial inactivated vaccine (type 1, strain NM01; TECON Biotechnology, Urumqi, Xinjiang, China); (2) E^rns^/E1/E2 mRNA-LNP group: subcutaneously immunized with 10 μg of E^rns^/E1/E2 mRNA-LNP formulated in PBS; (3) E2/IgG1 mRNA-LNP group: subcutaneously immunized with 10 μg of E2/IgG1 mRNA-LNP formulated in PBS; (4) PBS control group: subcutaneously immunized with 0.5 mL of sterile PBS. All groups received an identical booster immunization at 14 days post-primary vaccination (dpv). Blood samples were collected at 0, 14, 28 and 35 dpv for antibody detection. At 28 dpv, three mice from each group were euthanized by cervical dislocation, and spleens were aseptically collected for lymphocyte proliferation assays. At 35 dpv, all remaining mice were challenged by intraperitoneal injection of the BVDV-1a strain (10^8.37^ TCID_50_/mL). Body weight was measured daily, and clinical symptoms (including activity level, coat condition, and diarrhea) were monitored and recorded throughout the experimental period. At 5, 7 and 10 days post-challenge (dpc), three mice per group were euthanized, and tissue samples, including heart, liver, spleen, lung, kidney, jejunum, colon, mesenteric lymph nodes and blood, were collected for viral RNA load detection and histopathological analysis.

### 2.7. Virus Neutralization Assay

Collected serum samples were inactivated at 56 °C for 30 min. Sera were then serially diluted two-fold in cell culture medium (DMEM containing 2% fetal bovine serum, FBS) in 96-well cell culture plates, starting from an initial dilution of 1:2. An equal volume of BVDV strain (200 TCID_50_) was added to each well, and the mixture was incubated at 37 °C for 1 h in a humidified incubator with 5% CO_2_. Following incubation, 100 μL of the serum–virus mixture was transferred to wells containing confluent MDBK cell monolayers in 96-well plates, with four replicates per dilution. The plates were incubated at 37 °C with 5% CO_2_ for 96–120 h, and cytopathic effects (CPE) were observed daily under an inverted microscope. CPE was defined as the presence of characteristic morphological changes, including cell rounding, vacuolation, pyknosis, lysis, and detachment. The 50% inhibitory concentration (IC_50_) was calculated. IC_50_ is defined as the concentration of antibodies in serum required to achieve 50% inhibition of BVDV infection in vitro. Neutralizing antibody (nAb) titers were calculated using the Reed-Muench method.

### 2.8. Lymphocyte Proliferation Assay

Spleens collected from mice at 28 dpv were placed in sterile PBS on ice and gently dissociated through a 70 μM cell strainer (Corning, New York, NY, USA) using a sterile syringe plunger to obtain single-cell suspensions. The cell suspensions were centrifuged at 1200 rpm for 15 min at 4 °C, and the supernatant was discarded. Red blood cells were lysed by resuspending the cell pellet in 1 mL of erythrocyte lysis buffer (Solarbio, Beijing, China) and incubating for 20 min at 4 °C. Following centrifugation, the cells were resuspended in PBS and counted. The lymphocytes were adjusted to a concentration of 1 × 10^7^ cells/mL with RPMI 1640 medium containing 10% horse serum. A volume of 100 μL of the lymphocyte suspension was added to each well of a 96-well cell culture plate, following by the addition of 100 μL of medium containing the BVDV E2 recombinant protein (2.5 μg/mL) or 100 μL of medium alone as the negative control. Each sample was tested in duplicate. The plates were incubated in a cell incubator at 37 °C with 5% CO_2_ for 36 h. Subsequently, 20 μL of CCK-8 reagent (Absin, Shanghai, China) was added to each well, and the plates were incubated for an additional 2–3 h. The absorbance was measured at 450 nm. The stimulation index (SI) was calculated as the ratio of the mean absorbance of antigen-stimulated wells to that of unstimulated control wells.

### 2.9. Quantification of BVDV RNA by RT-qPCR

RT-qPCR for detection of BVDV RNA was performed using 2× SYBR Green qPCR Master Mix (Takara, Shiga, Japan) with the following designed primer pair: forward primer: 5′-GGGTAGCAACAGTGGTGAGT-3′ and reverse primer: 5′-CGGGCTAAGATGTGCTTTGG-3′. The qPCR reaction mixture consisted of 10 μL of 2× SYBR Green qPCR Master Mix, 0.4 μL of each primer (10 μM), 2 μL of cDNA template (prepared as described above), and 7.2 μL of RNase-free water, in a total volume of 20 μL. The amplification conditions were as follows: initial denaturation at 95 °C for 30 s, followed by 40 cycles of denaturation at 95 °C for 5 s and annealing/extension at 60 °C for 30 s. A standard curve was generated using 10-fold serial dilutions of a plasmid containing the BVDV 5′-UTR target sequence and was used to quantify the viral load in each sample. Viral loads were expressed as log_10_ viral RNA copies per mg of tissue or per mL of blood.

### 2.10. Pathological Tissue Sectioning

Tissue samples collected at necropsy were fixed with 4% (*w*/*v*) paraformaldehyde, dehydrated through a graded ethanol (70, 80, 90, 95 and 100%), embedded in paraffin wax, sectioned at 4–5 μM thickness using a microtome, stained with hematoxylin and eosin (H&E), and mounted on glass slides. Histopathological changes were observed and photographed under a light microscope.

### 2.11. Statistical Analysis

GraphPad Prism 10.0 was used for graphical and statistical analyses. Data are presented as the mean ± standard deviation (SD). One-way or two-way analysis of variance followed by Tukey’s multiple comparison test was used to compare differences among groups. Statistical significance was defined as follows: ns, *p* ≥ 0.05 (not significant); *, *p* < 0.05; **, *p* < 0.01; ***, *p* < 0.001; ****, *p* < 0.0001.

## 3. Results

### 3.1. Construction of BVDV mRNA Vaccine Candidates

**Figure 1:**
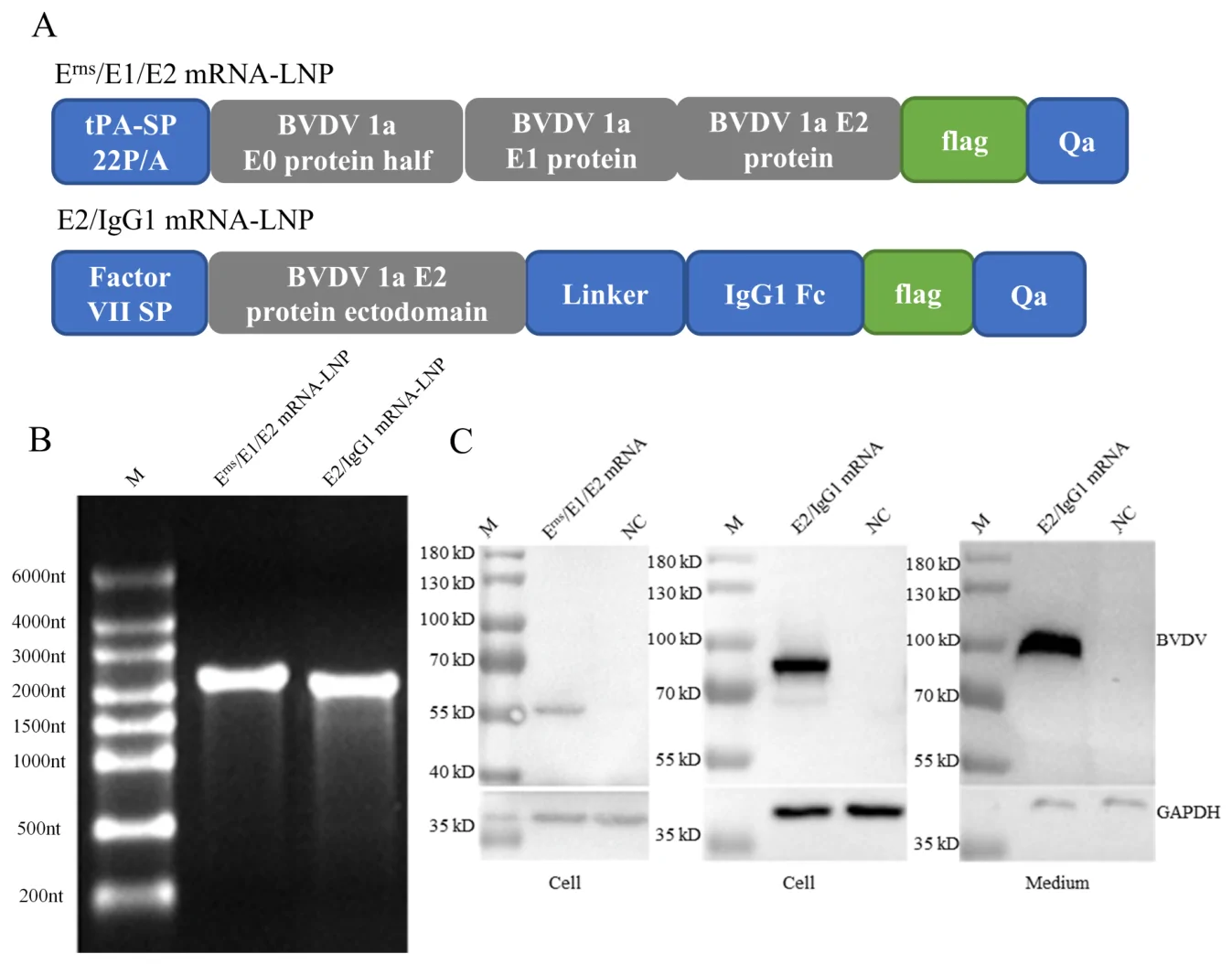
Construction of BVDV mRNA vaccine candidates. (**A**) Schematic diagrams of the two mRNA-based BVDV E protein expression constructs. The first construct (E^rns^/E1/E2 mRNA) encodes the tPA signal peptide (tPA-SP), a 22P/A linker, BVDV-1a E^rns^, E1, and E2 proteins in tandem, a C-terminal Flag tag, and a Qa sequence. The second construct (E2/IgG1 mRNA) encodes the Factor VII signal peptide (Factor VII SP), the BVDV-1a E2 protein ectodomain, a flexible linker, human IgG1 Fc domain, a C-terminal Flag tag, and a Qa sequence. Color coding: blue, signal peptide, linker, or tag; gray, viral protein; green, epitope tag. (**B**) Agarose gel electrophoresis of in vitro transcribed mRNA. M: RNA marker (200–6000 nt). (**C**) Western blot analysis of protein expression in HEK-293T cells transfected with naked mRNA. M: protein marker (35–190 kDa).

Two mRNA vaccine candidates targeting the BVDV envelope glycoproteins were designed and constructed: the E^rns^/E1/E2 mRNA vaccine encoding the full-length Erns-E1-E2 polyprotein and E2/IgG1 mRNA vaccine encoding the ectodomain of E2 protein fused to human IgG1 Fc segment (Figure 1A). Qa and the FLAG tag were incorporated into both vaccine constructs. Qa is a cis-regulatory element (Exin21, CAACCGCGGTTCGCG GCCGCT) encoding the heptapeptide QPRFAAA, which stabilizes mRNA and enhancing protein expression. A FLAG tag was included to facilitate verification of target protein expression by Western blot. The coding sequences of both vaccines were codon-optimized for enhanced expression in mammalian cells. The optimized coding sequences were cloned into the pUC57 plasmid, and mRNA was synthesized via in vitro transcription using linearized plasmids as templates. Following purification, mRNA integrity was confirmed by agarose gel electrophoresis, which showed distinct bands of the expected size with no visible degradation (Figure 1B). To verify protein expression, HEK-293T cells were transfected with each mRNA construct using Lipofectamine 3000, and protein expression was analyzed by Western blot at 24 h post-transfection using anti-E2 monoclonal antibody (Figure 1C). The E^rns^/E1/E2 mRNA group showed a distinct band corresponding to the E2 protein (55 kDa) in cell lysates. Because the E^rns^-E1-E2 coding sequence is translated as a single polyprotein, which is then processed into three individual native glycoproteins (Erns, E1 and E2) by host signal peptidases [13]. This band confirmed successful expression and processing of the polyprotein. For the E2/IgG1 mRNA group, bands corresponding to the E2-Fc fusion protein (70 kDa) were detected in both cell lysates and culture supernatants (Figure 1C). These results confirmed that both mRNA vaccine candidates could effectively express the target proteins in mammalian cells.

### 3.2. Preparation and Characterization of BVDV mRNA-LNP Vaccines

**Figure 2:**
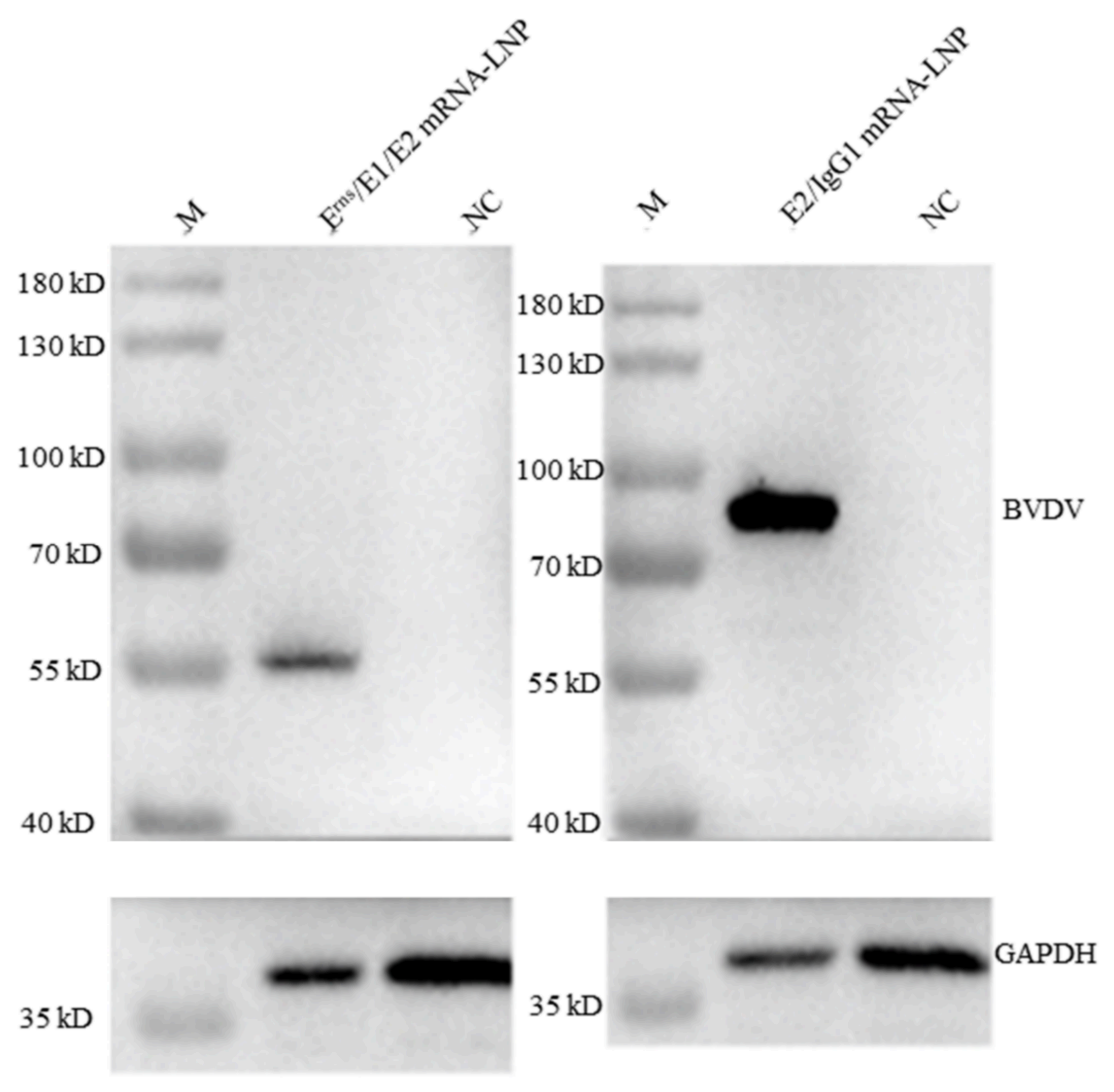
Protein expression of LNP-encapsulated mRNA in transfected cells. HEK-293T cells were transfected with LNP-encapsulated mRNA, and the E2 expression was detected by Western blot.

The sequence-verified mRNAs were encapsulated into LNPs using a microfluidic mixing system. The physicochemical properties of the resulting mRNA-LNP formulations, including average particle size, polydispersity index (PDI), and encapsulation efficiency (EE%), were characterized and are summarized in Table 1. Both mRNA-LNP formulations exhibited uniform particle sizes, with average diameters of 84.55 nm (E^rns^/E1/E2) and 85.57 nm (E2/IgG1), and low PDI values (<0.1), indicating homogeneous particle populations. The encapsulation efficiencies were 97.10% for E^rns^/E1/E2 and 97.63% for E2/IgG1, demonstrating efficient mRNA encapsulation by the LNP delivery system. To confirm that the encapsulated mRNA retained expression capacity, HEK-293T cells were transfected with each mRNA-LNP formulation, and E2 protein expression was verified by Western blot analysis. Both formulations directed robust and specific expression of the target proteins in transfected cells (Figure 2). These results indicated that the LNP encapsulation process did not compromise mRNA integrity or translational activity.

### 3.3. E^rns^/E1/E2 mRNA Vaccines Elicit Robust Immune Responses

**Figure 3:**
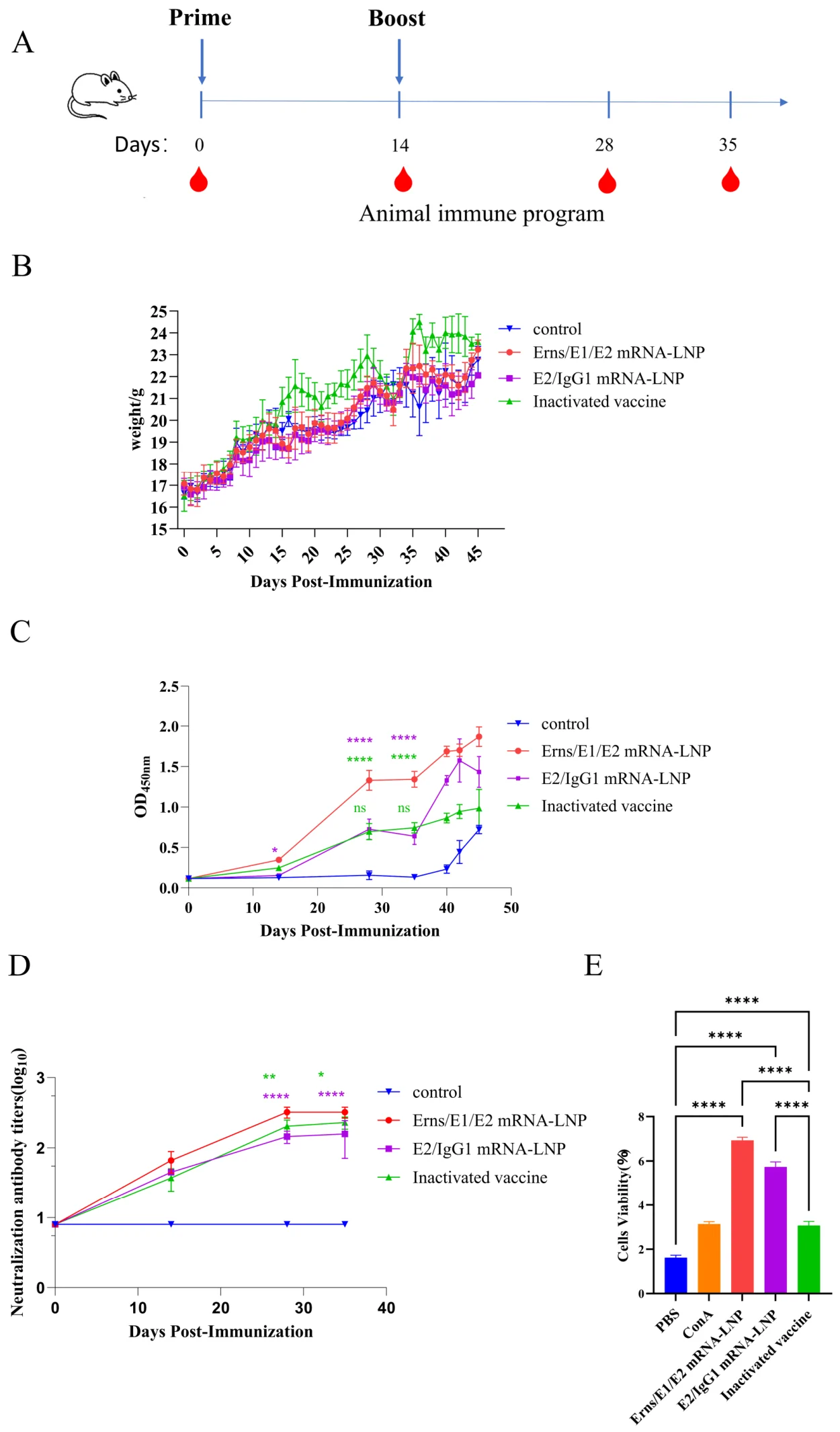
Immune responses elicited by BVDV mRNA-LNP vaccines in BALB/c mice. (**A**) Schematic diagram of the immunization protocol. (**B**) Body weight changes in BALB/c mice following primary immunization. (**C**) E2-specific antibody levels (OD_450_) measured by indirect ELISA in serum samples collected at the indicated time points post-immunization. (**D**) Neutralizing antibody titers (log_10_NT_50_) determined by virus neutralization assay at the indicated time points post-immunization. log_10_NT_50_ titers were determined by the Reed–Muench method from two-fold serial dilutions starting at 1:2; (**E**) Lymphocyte proliferation responses assessed by CCK-8 assay at 28 dpv. Splenocytes were stimulated with recombinant BVDV E2 protein, and the stimulation index (SI) was calculated. ns, not significant; *, *p* < 0.05; **, *p* < 0.01; ****, *p* < 0.0001.

The adaptive immune responses induced by the two mRNA vaccine candidates were evaluated in BALB/c mice and compared with those induced by a commercial inactivated vaccine (type 1, strain NM01). Serum samples were collected at 14, 28, and 35 dpv for analysis of BVDV-specific antibody responses and neutralizing antibody titers, as well as at 5, 7, and 10 dpc for post-challenge antibody kinetics. Splenocytes were collected at 28 dpv for analysis of cellular immune responses (Figure 3A). No significant adverse reactions were observed in any of the vaccine groups throughout the immunization period, and all mice exhibited steady weight gain comparable to that of the PBS control group, indicating a good safety profile of the mRNA vaccine candidates (Figure 3B).

**E2-specific antibody responses:** BVDV E2-specific antibody levels in serum were measured by indirect ELISA. Mice immunized with E^rns^/E1/E2 mRNA-LNP or the inactivated vaccine seroconverted as early as 14 dpv, with detectable E2-specific antibodies in all animals within these groups. In contrast, seroconversion was first observed at 28 dpv in mice immunized with E2/IgG1 mRNA-LNP. At 28 dpv (two weeks post-booster), mean E2-specific antibody levels (OD_450_) were 1.327 ± 0.124 in the E^rns^/E1/E2 mRNA-LNP group, which were 1.83-fold higher than those in the E2/IgG1 group (0.726 ± 0.126) and 1.91-fold higher than those in the inactivated vaccine group (0.694 ± 0.101). The E^rns^/E1/E2 mRNA-LNP group exhibited significantly higher antibody levels than both the E2/IgG1 mRNA-LNP group and the inactivated vaccine group at all post-booster time points tested (*p* < 0.0001 for all comparisons), with antibody levels continuing to increase until 35 dpv (Figure 3C).

**Neutralizing antibody responses:** Virus neutralization assays were performed to measure the titers of neutralizing antibodies induced by the different vaccines. Antibody titers (log_10_NT_50_) were determined by the Reed–Muench method from two-fold serial dilutions starting at 1:2. At 28 dpv (two weeks post-booster), mean neutralizing antibody titers (log_10_NT_50_) were 2.50 ± 0.082 in the E^rns^/E1/E2 mRNA-LNP group, significantly higher than those in the E2/IgG1 mRNA-LNP group (2.16 ± 0.082; *p* < 0.0001) and the inactivated vaccine group (2.30 ± 0.091; *p* < 0.01). At 35 dpv (three weeks post-booster), neutralizing antibody titers remained stable in the Erns/E1/E2 mRNA-LNP group (2.51 ± 0.082), which remained significantly higher than those in the E2/IgG1 mRNA–LNP group (2.16 ± 0.231; *p* < 0.0001) and the inactivated vaccine group (2.36 ± 0.091; *p* < 0.05). Consistent with the ELISA results, these data confirm that the E^rns^/E1/E2 mRNA–LNP vaccine elicited significantly stronger humoral immune responses than the other vaccine formulations (Figure 3D).

**Lymphocyte proliferation:** To evaluate cellular immune responses, splenocytes collected at 28 dpv were stimulated with recombinant BVDV E2 protein, and lymphocyte proliferation was assessed by CCK-8 assay (Figure 3E). The stimulation index (SI) of the E^rns^/E1/E2 mRNA-LNP group was 6.927 ± 0.145, which was significantly higher than that of the E2/IgG1 mRNA-LNP group (*p* < 0.0001) and the inactivated vaccine group (*p* < 0.0001). These results demonstrated that the E^rns^/E1/E2 mRNA-LNP vaccine elicited significantly stronger cellular immune responses than both control vaccines.

### 3.4. Protective Efficacy of BVDV mRNA Vaccines

To evaluate the protective efficacy of the vaccine candidates, all mice were challenged intraperitoneally with 1 mL of BVDV-1a (10^8.37^ TCID_50_/mL) at 35 dpv. Protective efficacy was assessed by monitoring clinical signs, detecting viral loads in various tissues and blood, and performing histopathological analysis of organ lesions at 5, 7, and 10 dpc. After challenge, mice in the unvaccinated groups commonly showed depression, huddling together, and rough and dull coats. In contrast, mice in the vaccinated group remained active, with smooth and neat coats, and no such abnormalities were observed.

**Figure 4:**
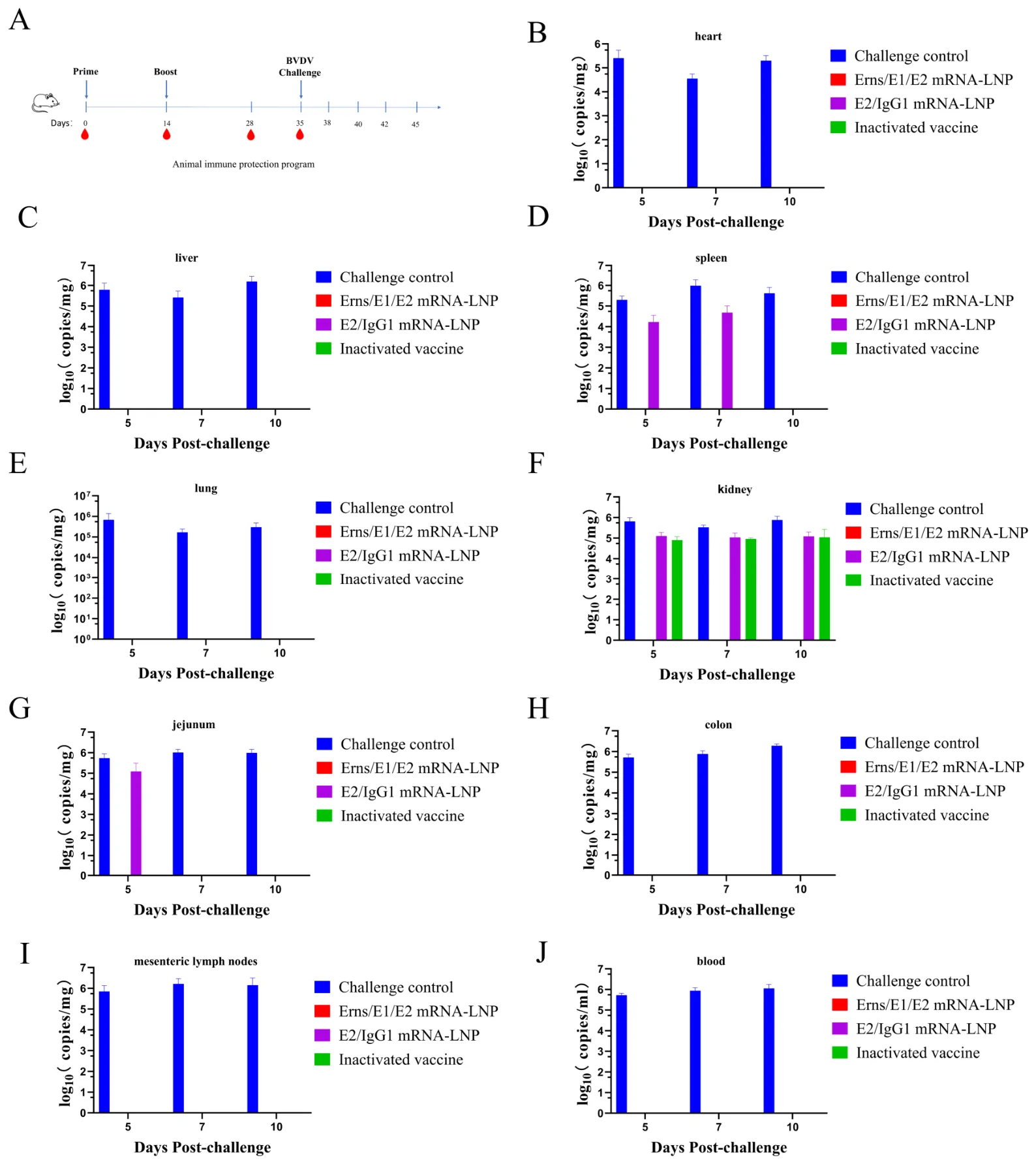
**Evaluation of protective efficacy of mRNA vaccines in mice.** (**A**) Schematic diagram of the immunization and challenge protocol. (**B**–**J**) Viral loads (log_10_ copies/mg tissue or copies/mL blood) in the heart (**B**), liver (**C**), spleen (**D**), lung (**E**), kidney (**F**), jejunum (**G**), colon (**H**), mesenteric lymph nodes (**I**), and blood (**J**) at 5, 7, and 10 dpc. Viral RNA was quantified by RT-qPCR. The dashed line indicates the lower limit of detection.

**Viral RNA load detection in tissues and blood (**Figure 4**).** In the challenge control group, viral RNA was detected in the heart, liver, spleen, lung, kidney, jejunum, colon, and mesenteric lymph nodes at 5, 7, and 10 dpc, with viral loads ranging from 10^4.55^ to 10^6.28^ copies/mg of tissue. Viral RNA was also detected in blood samples, ranging from 10^5.72^ to 10^6.04^ copies/mL.

In the E^rns^/E1/E2 mRNA-LNP group, no viral RNA was detected in any tissue (the heart, liver, spleen, lung, kidney, jejunum, colon, or mesenteric lymph nodes) or blood at any time point (5, 7 or 10 dpc), demonstrating that the E^rns^/E1/E2 mRNA vaccine conferred complete protection against BVDV challenge, with 100% viral clearance in all tested tissues and blood.

In the E2/IgG1 mRNA-LNP group, viral RNA was detected in the kidney at 5, 7 and 10 dpc, with viral loads ranging from 10^5.03^ to 10^5.10^ copies/mg. In the spleen viral RNA was detected at 5 and 7 dpc with viral loads ranging from 10^4.22^ to 10^4.68^ copies/mg, but undetectable at 10 dpc. In jejunum tissues viral RNA was detected at 5 dpc with viral loads of 10^5.09^ copies/mg, but not at 7 or 10 dpc. No viral RNA was detected in the heart, liver, lung, colon, mesenteric lymph nodes, or blood at any time point.

In the inactivated vaccine group, viral RNA was detected only in kidney tissues at 5, 7, and 10 dpc, with loads ranging from 10^4.91^ to 10^5.04^ copies/mg. No viral RNA was detected in the heart, liver, spleen, lung, jejunum, colon, mesenteric lymph nodes, or blood at any time point.

Collectively, no viral RNA was detected in the heart, liver, lung, colon, mesenteric lymph nodes, or blood of any vaccinated group (Figure 4), indicating that all three vaccine formulations provided varying degrees of protection against BVDV replication, with the E^rns^/E1/E2 mRNA-LNP vaccine achieving sterilizing immunity (Figure 4).

**Figure 5:**
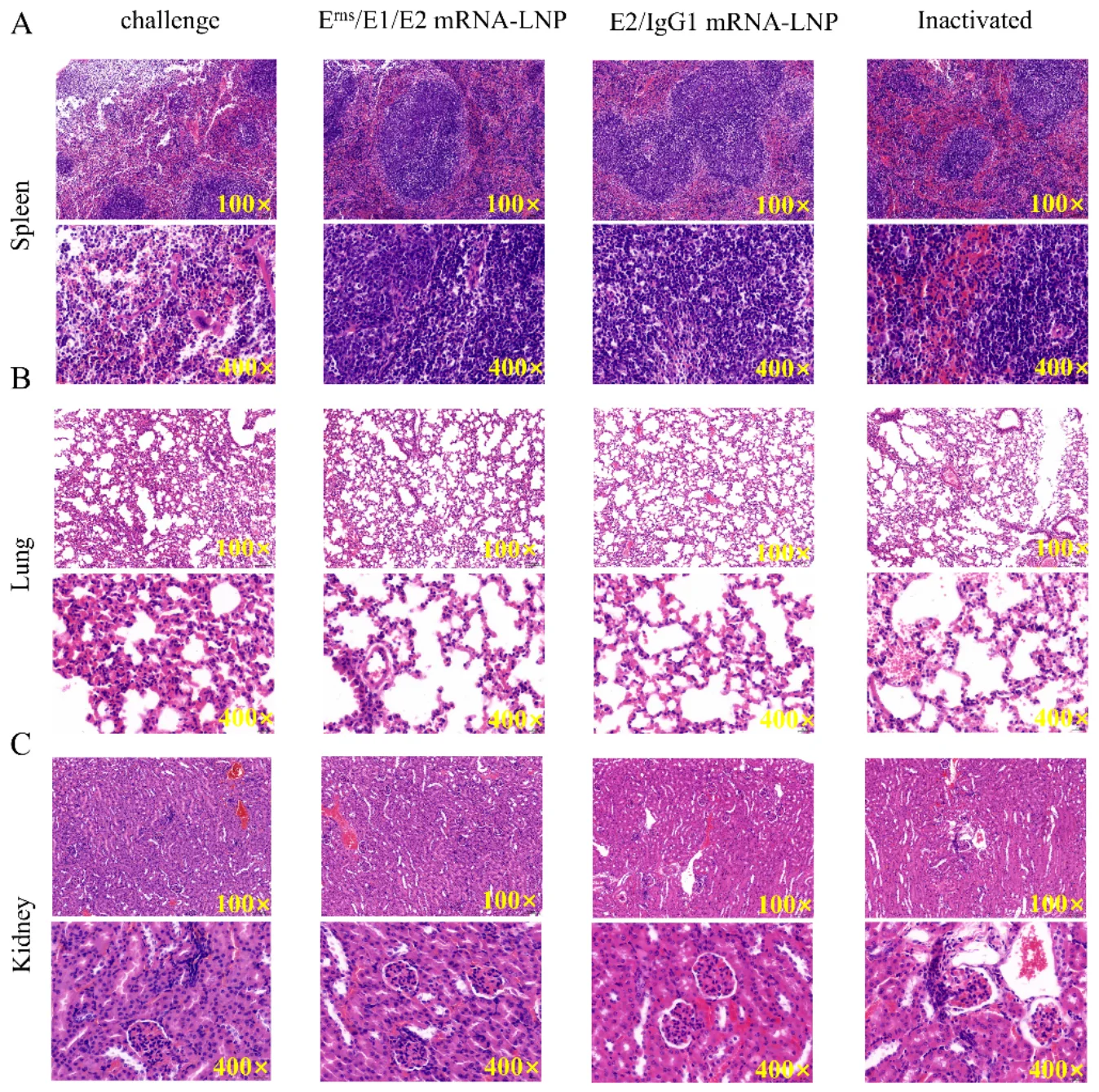
**Histopathological analysis of tissue sections from BVDV-challenged mice.** Representative H&E-stained sections of spleen (**A**), lung (**B**), and kidney (**C**) from mice in each group at 7 dpc.

**Histopathological analysis:** Mice in the challenge control group exhibited pronounced pathological changes in multiple organs following BVDV challenge. In the spleen, marked disorganization of splenic nodule was observed, including blurring of the boundary between the red pulp and white pulp, significant depletion of white pulp lymphocytes, infiltration of multinucleated giant cells, and foci of lymphocyte necrosis. Lung tissues showed extensive thickening of alveolar walls and inflammatory-cell infiltration. No significant histopathological abnormalities were observed in kidney tissues (Figure 5). In contrast, no significant histopathological abnormalities were observed in the spleen, lungs, or kidneys of mice immunized with E^rns^/E1/E2 mRNA-LNP, E2/IgG1 mRNA-LNP, or the inactivated vaccine, indicating that all vaccine formulations provided effective protection against BVDV-induced tissue damage.

## 4. Discussion

BVDV remains one of the most significant pathogens affecting the global cattle industry, causing substantial economic losses [26,27]. Vaccination is the primary strategy for BVDV control; however, conventional modified live and inactivated vaccines have significant limitations that hinder their effectiveness in eradication programs [28]. Modified live vaccines, while highly immunogenic, do not enable the differentiation of infected from vaccinated animals (DIVA), carry the risk of virulence reversion, and can cause persistent infection in vaccinated cattle [29,30]. In contrast, inactivated vaccines, although safer, often induce short-lived immune protection, necessitating frequent booster doses and failing to provide complete protection against heterologous strains [31]. These limitations highlight the urgent need for next-generation BVDV vaccines with improved safety, efficacy, and DIVA compatibility. mRNA vaccine technology has emerged as a transformative platform for vaccine development, as demonstrated by its unprecedented success in the fight against COVID-19 [32,33,34,35,36]. In this study, we demonstrated that the E^rns^/E1/E2 mRNA vaccine, encoding the E^rns^-E1-E2 polyprotein, induced robust humoral and cellular immune responses and provided complete protection against BVDV challenge in a mouse model, outperforming both the E2/Fc fusion mRNA candidate and a commercial inactivated vaccine.

Regarding vaccine design, two mRNA vaccine candidates were engineered based on the BVDV envelope glycoprotein sequences. The coding sequence of the E^rns^/E1/E2 vaccine encodes the full-length BVDV E^rns^, E1, and E2 proteins as a single polyprotein. A human tissue plasminogen activator signal peptide (tPA-SP, containing a 22P/A mutation) was introduced at the 5′ terminus to improve antigen expression efficiency. A Qa sequence was fused at the 3′ terminus to promote antigen expression and improve immune responses. The coding sequence of the E2/IgG1 vaccine is centered on the extracellular domain of the BVDV E2 protein. A human coagulation factor VII signal peptide (Factor VII SP) was used at the 5′ terminus to direct protein secretion, while the 3′ terminus encodes the human IgG1 Fc fragment fused via a flexible linker. The Fc fusion strategy is intended to enhance antigen uptake and presentation through Fc receptor-mediated pathways, thereby potentiating immune responses.

In terms of immunogenicity and protective efficacy, E^rns^/E1/E2 mRNA vaccine induced the highest levels of BVDV-specific cellular immune responses and specific neutralizing antibodies compared with both the E2/IgG1 mRNA vaccine candidate and the inactivated vaccine (type 1, strain NM01), indicating superior immunogenicity. BVDV challenge experiments further demonstrated that the E^rns^/E1/E2 mRNA vaccine conferred the most robust protection among all experimental vaccines. No viral RNA was detected in any tissue or blood samples at 5, 7 or 10 dpc, indicating complete viral clearance. In contrast, viral RNA was still detectable in the spleen, jejunum, and kidney of mice immunized with the E2/IgG1 mRNA vaccine at 5 dpc and in the kidney of mice immunized with the inactivated vaccine at all time points post-challenge. These results demonstrate that the viral clearance efficiency of the E^rns^/E1/E2 mRNA vaccine in mice is significantly superior to that of both control vaccines. The superior protective efficacy of the E^rns^/E1/E2 mRNA vaccine can be attributed to several key design features of the E^rns^-E1-E2 proteins. Importantly, this construct does not express a simple E^rns^-E1-E2 fusion protein; rather, the expressed polyprotein is co-translationally processed by host signal peptidases into three individual native glycoproteins, which then spontaneously assemble into higher-order structures within the endoplasmic reticulum [37,38]. Specifically, E1 and E2 form covalent E1/E2 heterodimers via a disulfide bond between Cys123 of E1 and Cys295 of E2, while Erns forms homodimers. The E1/E2 heterodimer is the functional unit mediating viral entry into host cells and harbors multiple conformational neutralizing epitopes that are absent in E2 alone, likely contributing to the induction of broader and more potent neutralizing antibody responses. E^rns^, in addition to serving as a target for neutralizing antibodies, possesses RNase activity that may further broaden the antigenic repertoire of the vaccine. The co-expression of all three envelope glycoproteins, processed and assembled into their native conformations, enables the E^rns^/E1/E2 mRNA vaccine to elicit both potent humoral and cellular immune responses, leading to the complete viral clearance observed in all tissues and blood. In contrast, the E2/IgG1 mRNA vaccine encodes only the E2 ectodomain and lacks the additional epitopes and native conformational context provided by E1 and Erns, which may explain its reduced immunogenicity and protective efficacy, as evidenced by residual viral RNA detected in multiple tissues post-challenge. These findings are consistent with previous studies demonstrating that vaccines incorporating multiple envelope glycoproteins induce more potent protective immune responses against pestiviruses than those based on E2 alone [39]. Although the Fc fusion strategy employed in the E2/IgG1 mRNA vaccine has been shown to enhance antigen presentation in some vaccine platforms, our results suggest that this benefit was insufficient to compensate for the loss of conformational epitopes and additional antigenic targets provided by the full-length E^rns^-E1-E2 polyprotein.

To our knowledge, this is the first study to directly compare the immunogenicity and protective efficacy of mRNA vaccines encoding different BVDV antigen designs. Our results are consistent with recent studies on mRNA vaccines for other veterinary pathogens, which have demonstrated the ability of mRNA platforms to induce robust and balanced immune responses at low antigen doses [40]. Importantly, the E^rns^/E1/E2 mRNA vaccine induced sterilizing immunity against BVDV challenge in mice, with no detectable viral RNA in any tissue or blood at all time points post-challenge. This level of protection is superior to that provided by the commercial inactivated vaccine, which showed residual viral RNA in the kidney even at 10 dpc. These findings highlight the potential of mRNA technology for the development of next-generation BVDV vaccines with improved efficacy over conventional formulations.

Despite these promising results, several limitations should be acknowledged. First, the experiments were conducted in a mouse model, which, while useful for initial immunogenicity and efficacy screening, does not fully recapitulate BVDV pathogenesis or immune responses in the natural bovine host. Further studies in cattle are required to confirm the vaccine’s efficacy, safety, and duration of immunity in the target species. Second, only a single BVDV-1a challenge strain was used in this study; future experiments should evaluate the cross-protective efficacy of the E^rns^/E1/E2 vaccine against heterologous BVDV-1 subgenotypes and BVDV-2 strains, which is critical for broad field applicability. Third, the long-term stability of the mRNA-LNP formulation under various storage conditions needs to be evaluated to assess its suitability for field deployment. Fourth, the DIVA capacity of the mRNA vaccine candidates was not assessed by testing antibodies against non-structural proteins (such as NS3), which are absent from the vaccine constructs and therefore from vaccinated animals. Additionally, dsRNA was not quantified during mRNA preparation, as only low levels of dsRNA are generated under the adopted IVT conditions; however, we acknowledge that dsRNA quantification would strengthen the characterization of our mRNA preparations. Therefore, future studies will address these limitations and further optimize the vaccine formulation and immunization regimen for application in cattle. 

## 5. Conclusions

In conclusion, this study successfully developed and evaluated two mRNA vaccine candidates against BVDV. The E^rns^/E1/E2 mRNA vaccine, encoding the E^rns^-E1-E2 polyprotein, induced significantly stronger humoral and cellular immune responses and provided complete immune protection against BVDV challenge in mice, outperforming an E2/Fc fusion mRNA candidate and a commercial inactivated vaccine. These results provide strong proof of concept for the development of mRNA-based BVDV vaccines and lay a solid foundation for further optimization and testing in the target animal species.

## Figures and Tables

**Table 1:** mRNA average size, polydispersity index, and encapsulation efficiency.

Formulation	Average Size (nm)	PDI	EE%
E^rns^/E1/E2 mRNA-LNP	84.55 ± 0.23	0.087 ± 0.006	97.10%
E2/IgG1 mRNA-LNP	85.57 ± 1.10	0.057 ± 0.030	97.63%

## Data Availability

All data generated for this study are included in the article.

## Abbreviations

The following abbreviations are used in this manuscript:

BVDV	Bovine viral diarrhea virus
ELISA	Enzyme-linked immunosorbent assay
LNP	Lipid nanoparticle
dpc	Days post challenge
dpv	Days post vaccination
CPE	cytopathic effects
IC_50_	50% inhibitory concentration
nAb	Neutralizing antibody
DIVA	differentiation of infected from vaccinated animals
